# Do All Opioid Drugs Share the Same Immunomodulatory Properties? A Review From Animal and Human Studies

**DOI:** 10.3389/fimmu.2019.02914

**Published:** 2019-12-12

**Authors:** Silvia Franchi, Giorgia Moschetti, Giada Amodeo, Paola Sacerdote

**Affiliations:** Department of Pharmacological and Biomolecular Sciences, University of Milano, Milan, Italy

**Keywords:** morphine, immunosuppression, fentanyl, buprenophine, oxycodone, methadone, tramadol, tapentadol

## Abstract

Suppression of the immune system has been constantly reported in the last years as a classical side effect of opioid drugs. Most of the studies on the immunological properties of opioids refer to morphine. Although morphine remains the “reference molecule,” other semisynthetic and synthetic opioids are frequently used in the clinical practice. The primary objective of this review is to analyze the available literature on the immunomodulating properties of opioid drugs different from morphine in preclinical models and in the human. A search strategy was conducted in PubMed, Embase, and the Cochrane databases using the terms “immunosuppression,” “immune system,” “opioids,” “Natural killer cells,” “cytokines,” and “lymphocytes.” The results achieved concerning the effects of fentanyl, methadone, oxycodone, buprenorphine, remifentanil, tramadol, and tapentadol on immune responses in animal studies, in healthy volunteers and in patients are reported. With some limitations due to the different methods used to measure immune system parameters, the large range of opioid doses and the relatively scarce number of participants in the available studies, we conclude that it is not correct to generalize immunosuppression as a common side effect of all opioid molecules.

Opioids remain the most effective and used drugs for severe pain treatment despite several negative aspects of poor health are often associated with their use. Moreover, opioids are the basic treatment for acute pain after surgery and for chronic pain, including cancer and non-cancer related pain. Among their well-known side effects, suppression of the immune system has been increasingly reported (1–3). In the last years the use of opioids increased and the concern that their immunological effects during and after surgery may impact on disease processes, such as bacterial, viral infections or cancer (2, 4), increased in parallel.

Moreover, opiates are also illicit drugs of abuse. The potential immunosuppressive effects of heroin and of the opioids used for opioid abuse treatment, such as methadone and buprenorphine, are of relevance since higher susceptibility to infection, or worst disease progression is reported in opioid addicted subjects (5, 6).

Numerous mechanisms at the basis of the effects of opioids on immune cells have been described. *In vivo*, the effects of morphine on immunity are mediated at both central and peripheral sites. Morphine binds to opioid receptors (OR) expressed on the cells of the immune system or on receptors within the nervous system. Opioid receptor activation in the central nervous system may modulate peripheral immunity via the hypothalamic pituitary adrenal axis and the autonomic nervous system (1–3, 7). This double modulation makes it somehow difficult to reconcile results obtained *in vitro* or *in vivo* with opioids, that often are different.

Most of the studies available on the immunological properties of opioids refer to morphine. Although morphine remains the “reference molecule,” other semisynthetic and synthetic opioids are frequently used in the treatment of pain in patients. It is therefore important to achieve a careful analysis of the different opioid drugs in order to understand whether they all display immunosuppressive properties. Although most data derive from preclinical studies, it is emerging that differentl opioids do not share the same immunosuppressive effects (1–3, 8).

The main objective of this review is to analyze the available literature on the immunomodulating properties of opioids drugs different from morphine. With this aim, we do not analyze in details the immune effects of morphine, since several excellent reviews have been published in recent years (1–3, 6–10). However, especially in the animal studies the effects of each opioid drug is often compared to that of morphine, and therefore the impact of morphine on immunity is indirectly reported. Figure 1 shows the structural formulae of the drugs considered in the present review.

**Figure 1:**
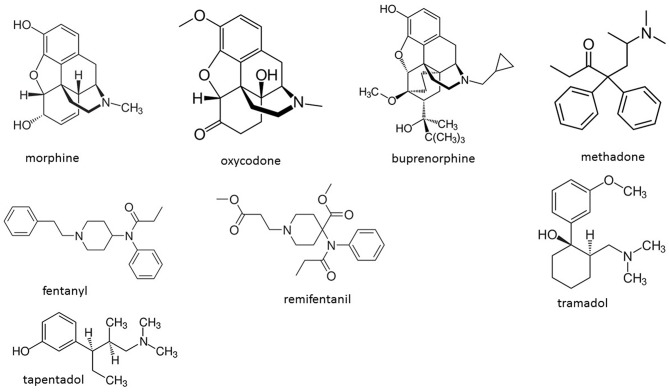
Structures of the opioid drugs described in the review. Oxycodone and buprenorphine are semisynthetic opioids; fentanyl, remifentanil, methadone, tramadol, and tapentadol are synthetic opioids.

In order to obtain the data, the databases Ovid MEDLINE (PubMed) and Embase (Ovid MEDLINE(R), Cochrane database and Web of Knowledge were searched using specific terms. To search for opioids, the terms used were: opioid OR opiate OR morphine OR buprenorphine OR methadone OR tramadol OR tapentadol OR oxycodone OR heroin OR fentanyl OR remifentanil. They were combined with a search for immunity: including immune^*^ OR Lymphocytes OR NK cell OR T cell OR cytokines OR immunosuppression. No limit for human or animal studies were added. All titles and abstracts were reviewed to assess their relevance for inclusion and reference lists from reviews and key publications were manually searched. Articles were also identified through searches of the authors' own files and previous reviews on the topic.

Two authors (PS and SF) performed literature searches and reviewed all titles and abstracts. Full papers were retrieved and the full texts analyzed by authors.

## Fentanyl

Fentanyl is a potent synthetic full agonist of the mu opioid receptor (MOR). It has a very short half-life and for this reason it has been for many years used mainly for the management of pain during surgery procedures. Only more recently the availability of a transdermal device allowed its use for chronic pain.

The effects of fentanyl on several immune parameters have been explored in animal and human studies after both acute and chronic treatment (1, 2, 7). Considering the wide use of this opioid in the perioperative period, several studies focused on its immunomodulatory effects at this time. This postoperative period is accompanied by immune suppression due to the interaction of several factors including analgesics used for pain treatment (1, 2, 11–13). An impaired immunity in the period may slow recovery, and may participate in the risk of developing infections and sepsis. Moreover, in cancer surgery, immunosuppression in the perioperative period is critical for the survival of cancer cells, due to the importance of the role of cell-mediated immunity in reducing micrometastatic formation (1, 2, 14, 15).

### Preclinical Studies

The immunopharmacological profile of fentanyl is similar to that of morphine. In preclinical studies, fentanyl has been reported to induce a dose-related immunosuppression (16). In rodents, continuous fentanyl infusion suppresses NK activity, lymphocyte proliferation, and cytokine production (16). Since NK activity is very important for the control of metastasis, several studies investigated the effect of fentanyl at doses clearly able to depress NK activity on the development of experimental tumor metastases (16–18). In these experiments animals were injected with a tumor cell line (MADB106 mammary adenocarcinoma) that is retained in the lung and grows as metastases. The cell tumor retention in the lungs inversely correlates with the efficacy of NK activity to destroy cancer cells. In these animals, it was shown that when fentanyl induced a dose-dependent decrease of NK cytotoxicity, the number of lung metastases increased (17, 18). Surgery and peri-surgery pain itself induces immunosuppression. Therefore, the effect of fentanyl on immune responses and metastasis was analyzed also following experimental surgery. While in two papers fentanyl and surgery-induced immunosuppression become additive (16, 19), in the work by Forget et al. (18) fentanyl did not worsen surgery-induced metastatic growth but did not prevent it. Molina-Martínez et al. (20) compared the effects of prolonged morphine and fentanyl treatment on macrophage function and TNF production. Both opioids significantly inhibited LPS-induced TNF production by macrophages at the same doses at which they produce antinociceptive effects. However, chronic opioid administration resulted in the loss of opioid-induced immunosuppressive effects indicating the development of tolerance. Interestingly, in the case of morphine, tolerance to the antinociceptive and immunosuppressive effects was parallel while in the case of fentanyl a significant analgesic effect was still present when the immunosuppressive effects had disappeared. The development of tolerance to the immunosuppressive effects of fentanyl has been reported also in Martucci et al. (16), suggesting that it is difficult to generalize the immunopharmacological properties of fentanyl since they may depend on dosage and length of treatment.

### Human Studies

The immunosuppressive properties of fentanyl have been consistently observed also in the human. The drug affects cellular immune responses and cytokine production in patients and healthy volunteers in a dose related fashion (1, 2, 21–24).

Most of the studies deal with fentanyl acute treatment during or after surgery.

The effect of high (75–100 μg/kg) or low (1–5 μg/kg) doses of fentanyl on natural killer cell cytotoxicity (NKCC) was assessed in 40 patients in the perioperative period (23). This study showed that both doses induced an inhibition of NK cytotoxicity on the first postoperative day; however, patients on the low dosage recovered faster, while patients on higher doses still showed reduction of immune parameters 48 h after surgery. These results were confirmed by Yardeni et al. (24), who studied the effect of high (70–100 μg /kg), intermediate (23–30 μg/kg), and low (2–4 μg/kg) doses of fentanyl on immune responses during the postoperative period in 60 patients. High and intermediate doses of fentanyl significantly depressed the levels of the proinflammatory cytokines IL-1 and IL-6 compared to patients on the low dose.

In a randomized controlled trial of 25 patients undergoing neck surgery, NK activity was assessed in patients treated with either fentanyl of flurbiprofen. In fentanyl treated patients NK cell cytotoxicity was suppressed more than in patients treated with flurbiprofen on day 1, but not day 2 post-operatively (25). More recently, the effect of fentanyl was assessed in 50 patients undergoing breast cancer resection who were given either propofol-remifentanil anesthesia with postoperative ketorolac analgesia or sevoflurane-remifentanil anesthesia with postoperative fentanyl for pain treatment (Sevoflurane-fentanyl group) (26). NK cytotoxicity was measured before and 24 h after surgery. In the sevoflurane-fentanyl group a significant inhibition of NK activity was measured, while no alterations were observed in the other treatment group. The authors also evaluated cancer recurrence or metastasis every 6 months for 2 years after surgery and did not find significant differences between groups.

Somehow in contrast with the suppressive effects of fentanyl on NK activity in patients, two healthy volunteer studies reported that acute intravenous fentanyl increased NK cell cytotoxicity, an effect that was shown to be due to an increase in the proportion of NK cells in the circulation rather than an increase in the cytotoxicity of individual NK cells (22, 27). The clinical significance of these observations remains to be understood.

## Methadone

Methadone is synthetic 3,3-diphenylpropylamine opioid with a unique pharmacological profile. It primarily acts at the MOR but also activates kappa (KOR) and delta (DOR) receptors. Moreover, it also binds N-methyl-D-aspartate receptor as a weak antagonist (28). Methadone is a potent and long-acting opioid and its PK and PD characteristics have made it the most used opioid for substitution therapy in opioid dependence (29). However, methadone is also a valuable drug in the management of chronic pain especially in cancer pain. Its efficacy as an NMDA antagonist suggests its use in neuropathic pain as well (28).

A small number of studies analyzed the impact on immunity of methadone in the experimental animal, while a few papers reported the immune status of opioid addicted subjects on methadone maintenance therapy in comparison to heroin abusers. No studies were found on the immune profile of methadone in chronic pain patients.

### Preclinical Studies

Old studies from the nineties of the last century showed that chronic methadone treatment in rats did not affect either *Trichinella* nor *Listeria* infection (30, 31), in contrast to chronic morphine treatment that significantly augmented infection. No effect was observed on antibody production after methadone treatment. Indeed a paper by Van De Laan (32) indicated that methadone prolonged treatment increases cellularity of spleen and lymph-nodes.

Methadone is administered as a racemic mixture of (R)-(-)- and (S)-(+)-enantiomers, with only (R)-(-)-methadone possessing opioid receptor agonist activity. Hutchinson et al. (33) investigated the specific immunomodulatory effect of the (R)-(–)–or(S)-(+)-enantiomer *in vivo* administration to mice and found that the (S)-(+)-enantiomer that is devoid of analgesic efficacy caused significantly greater inhibition of lymphoproliferation than the (R)-(_)- or racemic methadone. The authors suggested that methadone-induced immunomodulation was not a classical opioid response but might be mediated by “non classical,” not yet identified, opioid receptors at central level.

The effect of methadone-prolonged treatment on macrophage functionality and its ability to influence contact hypersensitivity and antibody production was studied by Filipczak-Bryniarska (34). From this work, that compared several opioids, it emerged a decrease of macrophage function, alteration of antibody production and of contact hypersensitivity in methadone treated mice. However, the percentage of inhibition was lower than that of morphine and fentanyl.

Interestingly methadone was found to protect mice from experimental autoimmune encephalomyelitis (35). The drug reduced clinical signs of the disease, the level of inflammatory cytokines produced by T cells and recruitment of inflammatory cells into the spinal cord. The authors conclude that opioid receptor signaling may be beneficial in the context of autoimmune neuroinflammation (35). This paper suggests therefore that the mild immunosuppressive activity of methadone may be exploited in autoimmune diseases.

In conclusion, from the preclinical studies reported methadone exerted a weak immunosuppressive activity.

### Human Studies

#### In vitro

Methadone was included in a study that compared the effect of several opioids added *in vitro* to human cells and that evaluated neutrophil and monocyte phagocytosis and oxidative burst responses, NK cell cytotoxicity and T cell activation *in vitro* (36). Confirming what was observed in the animal experiments, in contrast to the morphine and fentanyl effects, *in vitro* methadone did not influence monocyte and neutrophil phagocytosis. None of the drugs tested modified NK activity, but methadone significantly decreased IL-6 production by T lymphocytes. As already reported above, the *in vitro* experiments are only partially representative of opioid effects *in vivo*, due to the fact that some effects are mediated by the activation of the MOR in the central nervous system.

Morphine and methadone are often used for treatment of neonatal abstinence syndrome (NAS). Therefore, Chavez-Valdez et al. (37) analyzed whether clinically relevant concentrations of different opioids may modify cytokine levels in cultured whole blood from preterm and full-term infants. All three MOR, KOR, DOR genes were expressed in mononuclear cells from preterm and full-term infants. Morphine and methadone, but not fentanyl, decreased LPS stimulated IL-1b, IL-6, IL-10, IL-12p70, and TNF.

Finally, in the elegant work by Borner (38) *in vitro* methadone was reported to increase the production of the Th2 cytokine IL-4, differently from morphine and buprenorphine. In this paper the authors identify the transcription factor NFAT and AP1 at the basis of methadone-induced IL-4 stimulation. Moreover, methadone and fentanyl were able to efficaciously induce MOR internalization on a T cell line (Jurkat T cells), while buprenorphine and morphine did not. Therefore, the authors are the first to suggest that the immunomodulating properties of the different opioids may depend on their ability to activate different signaling pathways after MOR stimulation.

#### In vivo

The hypothesis that significant alterations of cellular immunity in heroin abusers might be normalized by switching to long-term methadone treatment was proposed several years ago in a first paper that presented the genetic damage provoked by different opioids in T lymphocytes (1, 39). Subsequent studies evaluated other immune responses, such as NK cytotoxicity, T cell subset number and function and phagocyte activity in patients under methadone maintenance treatment in comparison with heroin abusers (40, 41). The studies also tried to analyze whether the improvement of immune responses observed with methadone treatment was dependent on the drug itself or on the modification of life style that may intervene during the maintenance treatment. A randomized clinical trial reported that the switch to methadone or buprenorphine treatment restored the immune function depressed by heroin in addicted individuals (40). Moreover, a controlled methadone or buprenorphine therapy also normalized the Th1/Th2 balance that was significantly unbalanced during chronic heroin use (41). The beneficial effect of methadone maintenance on immune system responses of heroin abusers has been consistently reported also more recently (42–44). Participation in methadone maintenance treatment was protective against hepatitis C incidence among illicit drug users and methadone exerted a dose-response protective effect on hepatitis C incidence (42). Naïve HIV-infected individuals using heroin and receiving methadone opioid substitution or controls (who never used opioids) were studied in the paper by Meijerink (44). Whole blood obtained from the two groups was stimulated with *Mycobacterium tuberculosis, Candida albicans*, and LPS and cytokine production was determined. The cytokine production stimulated with LPS was significantly down-regulated in HIV-infected heroin users while in methadone users cytokine response was not different from subjects who never used opioids. Similarly, methadone treatment was able to re-establish the number and the expression markers of dendritic cells that were significantly altered in a cohort of heroin drug abusers (43). Surprisingly in another work, heroin abusers on methadone maintenance treatment had IL-1β, IL-6, and IL-8 levels significantly higher than in control healthy subjects. In addition, a significant correlation was observed between the plasma TNF-α and IL- 6 levels, the dose and the duration of methadone maintenance (45). The authors suggest that methadone maintenance treatment may induce long-term systemic inflammation.

Several factors may participate in the immune system amelioration, such as the different immunopharmacological profiles of the opioids used, the absence of withdrawal episodes, and a safer use of contaminated drug syringes or other instruments. Unfortunately, due to the paucity of controlled longitudinal epidemiological studies, based on the available data it is not possible to reach an answer whether opioid-induced immunosuppression or behaviors associated with drug abuse may be responsible for higher incidence of infections in addicted patients.

## Oxycodone

The semisynthetic opioid oxycodone is one of the most prescribed in Europe and the United States both for acute and chronic pain and has been recently recognized as abused drug. Oxycodone is a relatively selective MOR agonist, its affinity for the MOR is less than that of morphine or methadone but it exerts a similar antinociceptive effect. This discrepancy has been explained on the basis of the pharmacokinetic properties of the molecule that passes the blood brain barrier easily. The analgesic effect is due in large part to the parental molecule itself. However, oxycodone is biotransformed in the liver by cytochrome into active metabolites with higher MOR affinity that participate in its analgesic efficacy (46).

Considering the elevated number of patients who had received oxycodone it is surprising the paucity of works concerning the immunomodulating effect of the drug. However, the papers published consistently report that this molecule has only a minimal impact on immunity, which in all cases is much lower than that of morphine or fentanyl.

### Preclinical Studies

The first evidence of the neutral immunopharmacological profile of oxycodone comes from the paper by Sacerdote et al. (47), a structure-related activity study that compared *in vivo* the impact of natural and semisynthetic opioids on lymphoproliferation, IL-2 production, and NK activity in the mouse. The data indicate that the C6 carbonyl substitution and the presence of a C7-8 single bond, like in oxycodone, potentiates the antinociceptive effect, but abolishes immunosuppression.

In a series of papers the *in vivo* treatment with oxycodone on macrophage functionality was studied (48, 49). Although not completely devoid of effect, oxycodone expressed weaker immunomodulatory properties than morphine and the authors concluded that oxycodone seems to be a safer opioid for chronic therapy. However, in one single study conducted *in vitro* with mouse splenocytes (50), an inhibitory effect of oxycodone was reported on lymphoproliferation with an inverted bell shaped curve, with only few intermediate concentrations active. Indeed the limitation of the study remains the fact that the *in vitro* experiments are only partially representative of opioid effects *in vivo*, due the to the fact that some effects are mediated by the activation of the MOR in the central nervous system.

### Human Studies

Boland (36) compared the effect of several opioids added *in vitro* to human cells and evaluated neutrophil and monocyte phagocytosis and oxidative burst responses, NK cell cytotoxicity and T cell responsiveness. Oxycodone did not influence monocyte and neutrophil phagocytosis nor NK activity, but it decreased IL-6 production by T lymphocytes. As reported above, oxycodone has an active metabolite that participates in the analgesic effect. Obviously in the *in vitro* studies this component is not present, limiting in part the significance of the results.

Only three studies were found that tried to assess the potential immunomodulatory effect of oxycodone in patients. General immune responses and local responses in the surgical wound were measured in children who underwent surgery (51). Bolus doses of diclofenac intravenously and rectally, continuous i.v. oxycodone infusion or continuous epidural infusion of bupivacaine—fentanyl were applied for pain treatment. The authors conclude that all post-operative pain treatments had similar effects on systemic and local immune responses with minor, probably clinically irrelevant differences.

Suzuki et al. (52) performed a retrospective study that analyzed the correlation between morphine or oxycodone administration and the presence of infections in patients with cancer pain. In this work no measurement of immune functionality was indeed present. This study enrolled 841 patients receiving one opioid continuously for more than 10 days. A significant higher number of patients treated with morphine developed infections in comparison to patients with oxycodone. These results indirectly suggested that morphine- induced immunosuppression may participate to infections in patients with cancer pain. More recently, a clinical study evaluated the effect of oxycodone hydrochloride injection on the immune responses of patients who underwent resection of rectal cancer under general anesthesia (53). At the end of surgery, patients were injected with 5 mg of oxycodone or with 5 mg of morphine and the number of T cell subsets and NK cells was assessed by flow cytometry. Both opioids exerted inhibitory effects on immune function but oxycodone had a smaller effect than morphine and the immunosuppression was short lasting, since the responses normalized by 6 h after treatment.

In conclusion from the scarce studies available the immunosuppressive effects of oxycodone, although present, appears always weaker than those of morphine and fentanyl.

## Buprenorphine

Buprenorphine is a semisynthetic thebaine derivative acting as a partial agonist of MOR, an ORL-1 full agonist and KOR and DOR antagonist. Buprenorphine has a high affinity for the opioid receptors, low intrinsic efficacy and it is characterized by a slow receptor binding kinetics. Due to the complex profile of this molecule at opioid receptors, its pharmacological profile is often different from the other opioid agonists (54). The molecule in transdermal patch is used for the treatment of chronic pain. Moreover, in the last years it is increasingly used for maintenance treatment of heroin addiction as an alternative to methadone (55).

### Preclinical Studies

Experimental animal work conducted by several research groups points to a safe profile of buprenorphine on immune responses when administered both acutely and chronically. Gomez Flores and Weber (56, 57) had shown that in the rat after the acute injection of equianalgesic doses of buprenorphine and morphine into the mesencephalic peri-acqueductal gray (PAG), buprenorphine did not alter NK cell cytotoxicity, T cell and macrophage function, while morphine significantly suppressed these functions. Similar results were also reported after continuous buprenorphine delivery in comparison with fentanyl (16). In a model of surgery stress in the rat, buprenorphine prevented all the biochemical, endocrine and immune modifications caused by pain, differently from the clearly evident fentanyl induced immunosuppression in the same experimental model (19).

The final effect of opioid drugs on immunity depends on several aspects: the intrinsic immunosuppressive property of the drug and the prevention of pain and neuroendocrine activation. Pain is a physical and psychological stressor that causes activation of the HPA axis and the sympathetic nervous system and can have a negative impact on immunity (58). In a rat model of experimental surgery the presence of post-operative pain increased corticosterone levels, decreased NK cytotoxicity and facilitated the spreading of metastasis of the NK-sensitive tumor MADB106 (19, 57). Morphine and fentanyl did indeed relieve pain, but also stimulated the HPA axis, decreased NK activity and did not prevent surgery-induced metastasis. In contrast, equianalgesic doses of buprenorphine prevented the HPA activation and immune system depression and controlled the increased number of tumor metastasis (19).

### Human Studies

Very few studies addressed the impact of buprenorphine on immune parameters in the human. In *in vitro* experiments using cells from human volunteers, buprenorphine had only limited effects on neutrophil, monocyte phagocytosis and oxidative burst and did not modify cytokine production (36). Recently two elegant studies (59, 60) showed the ability of buprenorphine to reduce CCL2-induced chemotaxis and transmigration into the brain of a sub class of monocytes, the CD14^+^CD16^+^ monocytes that play an important role in HIV sustained neuroinflammation in AIDS patients. In these papers the authors also demonstrate the presence of functional MOR and KOR on this monocyte subtype. The clinical relevance of these results is positively discussed by the authors, who suggest the possibility that in HIV-opioid addicted patients, buprenorphine maintenance treatment may help to prevent neuroinflammation and neurological dysfunction observed in AIDS patients.

Two studies are published on the effect of buprenorphine treatment *in vivo* on immunity in humans, recruiting drug abusers who were on chronic maintenance with the drug for opioid dependence (40, 41). Although buprenorphine doses were quite high (mean dose 9.3 ± 2.3 mg/day), a significant amelioration of immune parameters was registered in comparison with those measured before starting the buprenorphine treatment. However, as already discussed for methadone, several factors may contribute to the positive effect on immunity, besides the immunopharmacological profiles of the different drugs, such as the control of withdrawal episodes and the change of habits linked to intravenous injections.

## Remifentanil

Remifentanil is an ultra-short-acting MOR agonist used in general anesthesia since it efficaciously and rapidly controls the autonomic, hemodynamic and somatic responses to noxious stimuli. Due to its molecular structure, blood esterases hydrolyze it, resulting in fast metabolism, and fall in serum concentrations after interruption of the infusion (61). The definition of its immunomodulating properties in the perioperative period could be of particular interest.

### Preclinical Studies

Sacerdote et al. (62) evaluated the effects of remifentanil continuous infusion on immune function in the rat and reported suppression of the immune response, characterized by a significant reduction of NK cytotoxicity, lymphocyte proliferation, and cytokine secretion. In another rat study remifentanil infusion also significantly reduced activation and cytokine production from broncho-alveolar neutrophils and macrophages in LPS induced lung injury (63).

### Human Studies

In accordance with what was observed in the rat, the *in vitro* addition of remifentanil to human neutrophils from healthy volunteers induced a dose dependent reduction of proinflammatory cytokine production (64, 65). In contrast low-dose (0.02–0.04 μg/ kg/min) remifentanil infusion in healthy volunteers did not cause any significant alteration in the number nor the cytotoxicity of NK cells after an 8-h infusion (66). These results once more suggest that depending on the cell type, the parameters and the *in vivo* or *in vitro* administration, the effect of opioids on immunity may be very different. Finally, cytokine secretion was reported to be reduced by remifentanil also in patients who underwent coronary artery bypass graft surgery. Interestingly in this study remifentanil (0.3–0.6 μg/kg per min) suppressed mainly proinflammatory cytokines such as IL-6, TNF, and IFN-γ, but did not significantly affect the anti-inflammatory cytokine IL-10; the authors suggest that remifentanil infusion may blunt the inflammatory response that may take place after cardiac surgery with cardiopulmonary bypass (67).

## Tramadol

Tramadol is an opioid drug with pharmacodynamics characteristics distinct to those of the classic opioids. It is a weak opioid that also blocks serotonin and noradrenaline reuptake. The tramadol metabolite M1 binds MOR with low affinity, while the parental molecule inhibits serotonin and noradrenaline uptake causing the activation of descending inhibitory monoaminergic pathways (68). Its effects on immunity were analyzed in preclinical and clinical studies with consistent results (1).

### Preclinical Studies

Tramadol did not induce any immunosuppressive effect either after acute or chronic treatment. When administered acutely to normal animals tramadol induced a clear immunoenhancing effect on several immune parameters such as NK cytotoxicity, proliferation of lymphocytes and cytokine production (69). In the animal studies, using specific antagonists, it was demonstrated that the immunostimulating activity of tramadol has to be ascribed to its serotoninergic activity (70).

The effects of tramadol on immunity was thereafter evaluated in different animal models of pain in direct comparison with morphine. In a rat model of neuropathic pain, tramadol did not affect the NK activity, while morphine depressed it (71). As already reported, pain associated with surgery is one important factor that participates in surgical stress-induced immunosuppression, and in particular NK activity is extremely sensitive to peri-operative stress (11, 58). Equi-analgesic doses of tramadol and morphine were studied in a preclinical model of surgery-induced suppression of NK activity; only tramadol prevented the reduction of NK cytotoxicity (72). Both tramadol and morphine similarly relieved pain, but tramadol also possessed intrinsic immunostimulating properties, that helped to protect NK activity (72).

### Human Studies

This profile of tramadol was confirmed also in human studies (73–75).

In *in vitro* studies, tramadol did not affect human polymorphonuclear activity from healthy volunteers (75).

The impact of morphine or tramadol on the immune function was studied in patients who underwent surgery and were treated for post-operative pain (73). In morphine treated patients, a prolonged reduction of immune function was measured. In contrast, patients treated with tramadol at equianalgesic dose with morphine showed a faster and full recovery of immune parameters depressed by surgery (73). The good profile of tramadol on immunity was reported in a different trial where morphine and tramadol were again given to provide analgesia after surgery (74).

## Tapentadol

Tapentadol is a novel, centrally acting analgesic drug used for treating moderate-to-severe pain (76). It is characterized by a dual mechanism of action; it binds with low affinity to MOR as an agonist and also inhibits noradrenaline reuptake (77). Both mechanisms contribute synergistically to its analgesic effect, thus resulting in analgesia with less opioid related side effects.

Only one study examined the impact on immune responses of this opioid in the mouse in comparison to morphine (78). In normal animals, consistently with what would be expected for the lower affinity of tapentadol for MOR, both the acute and chronic administration of the opioid did not affect lymphocyte or macrophage cytokine production, whereas morphine decreased all cytokines measured. The modulation by morphine and tapentadol of peripheral cytokine production was evaluated also in mice suffering from chronic pain consequent to sciatic nerve ligation. The presence of chronic pain itself had a negative impact on cytokines. Both morphine and tapentadol exerted a similar and satisfactory analgesic activity, but only in tapentadol treated mice a significant restoration of the anti-inflammatory cytokines was present. The authors concluded that acute and chronic tapentadol is neutral on cytokine production, and hypothesized that the synergy of the two mechanisms of action of tapentadol, which play an important role in analgesia, is not relevant for the immunosuppressive properties.

Differently from tramadol, tapentadol did not enhance any immune parameter, confirming that the immunostimulating activity of tramadol depends mainly on the serotoninergic mechanisms.

### Human Studies

We could not find any clinical work examining the effects of tapentadol on immunity.

## Conclusions

While for decades opioids have been considered as a class of drugs with similar clinical and side effects, in the last years it is increasingly emerging that the different molecules have peculiar characteristics that differentiate them. The immunosuppressive activity is certainly one of the side effects that more than others differentiates opioids. The mechanisms at the basis of these differences need to be studied in detail and at the moment only suggestions or hypotheses can be put forward. The many observations reported of different results obtained when the opioids are added *in vitro* or administered *in vivo* point to the importance of both a direct effect mediated by opioid receptors expressed by immune cells and indirect effects due to MOR activation in the nervous system (1–3, 10).

What has been well-demonstrated in a series of animal studies is that in the absence of MOR the immunosuppressive effect of morphine is lost (10, 79, 80). This fact may explain why opioids with low opioid receptor affinity such as tramadol or tapentadol have a lower immunosuppressive activity in animal and human studies at analgesic doses. Their analgesic effect is sustained by the combination in the same molecule of more mechanisms of action (such as opioid receptor binding and monoaminergic stimulation), but the additive/synergistic mechanisms that are important for analgesia do not have a role in the immunosuppression.

However, this explanation cannot be applied to other potent opioids such as buprenorphine, oxycodone or methadone. The recent advances in the comprehension of opioid pharmacology and the biology of the opioid receptors and their signaling (81, 82) pathways may help to investigate the reasons for these differences. The term “biased agonism” (or functional selectivity) refers to the ability of different ligands of the same receptor to stabilize the receptor in different active states. This different stabilization may lead to the activation of different intracellular pathways: a biased agonist preferentially activates one signaling pathway rather than another (83, 84).

Biased agonists of GPCRs, such as those binding opioid receptors, might activate G protein-mediated pathways while other agonists might involve β-arrestin-2. It has been suggested that analgesia is associated with G-protein pathways, while arrestin recruitment with some opioid-related adverse effects (85). Although this aspect is still under debate, some studies have tested this paradigm evaluating respiratory depression, gastrointestinal effects or abuse potential, but the impact of G-protein vs. arrestin activation in immunosuppression has never been explored. For example unlike opioids such as morphine, fentanyl and methadone, buprenorphine does not recruit β-arrestin to the receptor (86) and as described above buprenorphine has been reported to be devoid of immunosuppressive properties. Indeed morphine is a balanced agonist, and its ability to recruit arrestin after receptor binding is lower than that of fentanyl or methadone that have been suggested to be a β-arrestin biased compounds (85, 87). However, morphine and fentanyl have comparable immunosuppressive activity, while methadone is a weaker immunomodulator and therefore the concept of biased agonist is not sufficient to explain differences.

As it is well-known, methadone is characterized by unique and diverse pharmacologic properties, including NMDA receptor antagonism, inhibition of serotonin and noradrenaline uptake and affinity for DOR in addition to MOR (28, 87). The possible role of these different mechanisms in the mild immunomodulation induced by methadone has never been assessed.

A further different aspect is represented by the human studies conducted in the heroin addicted subjects switched to methadone or buprenorphine maintenance. In this situation several variables may be involved. Short acting opioid drugs such as morphine and heroin have been shown to deeply affect immune system (1, 41) while long acting opioids, such as methadone and buprenorphine, can progressively restore immune function and cytokine levels (40). An interesting point to be considered is the observation that during opioid withdrawal several biochemical and hormonal perturbations are present. These alterations or fluctuations of hormones and neurotransmitters may be implicated in opioid-induced immunosuppression (88, 89). It is therefore possible that the re-establishment of immune responses that is present with methadone and buprenorphine in contrast to heroin could partially depend on the constant activation of MOR. Consistently with this hypothesis, in a monkey model of HIV infection the administration of morphine according to a protocol that prevented withdrawal did not have a negative impact on immunity and did not worsen HIV disease progression (90). In contrast, the same authors also demonstrated that an abrupt discontinuation of opioids precipitated immune dysfunction (91). It can be hypothesized that the differences in the immune response observed in drug addicted subjects could be attributed to the controlled methadone or buprenorphine use pattern that reduced withdrawal-induced stress (92). Moreover, long lasting treatments with methadone and buprenorphine are able to normalize the HPA axis that is altered in heroin abusers (93, 94). The normalization of the HPA axis could play an additional role in restoring the altered immune function present in heroin addicts.

Finally, it must be remembered that besides pharmacodynamic also pharmacokinetic differences may be important for the diverse immune effects of opioids. For example morphine and oxycodone possess active metabolites that bind MOR, while fentanyl and methadone do not. Indeed a few studies have demonstrated that morphine-glicuronide metabolites exert immunosuppressive activity both in the animal and in cancer patients (95, 96). Considering that during chronic opioid treatment metabolites can accumulate also this aspect deserves further attention.

In conclusion, evidence from preclinical, healthy volunteer and clinical studies suggests that different opioids have a variable impact on immunity (Table 1). Indeed only morphine, fentanyl and remifentanil are consistently described as immunosuppressive both in the animal and in the human. All other opioid molecules have been found to have a weaker impact on immunity. In particular convincing data have been gathered concerning the neutral effect on immunity of tramadol and buprenorphine. While for tramadol the lack of immunosuppressive activity can be due to the lower MOR affinity combined with the serotoninergic component, the reasons for the profile of buprenorphine are not yet clear and only speculative. The data available on the potential immune effects of oxycodone are really too few to draw a general conclusion on this molecule, although some differences from morphine are present. As far as methadone is concerned, it appears from animal studies that it may have intrinsic immunosuppressive properties; however when used as maintenance treatment in heroin addicted subjects it can restore immune function. As reported above several variables may be involved in these beneficial effects. Unfortunately no study on methadone for chronic pain treatment is available.

**Table 1:** Summary of the effects of opioid drugs on immunity.

	**Preclinical studies**		**Human studies**	
	***In vitro***	***In vivo***	***In vitro***	***In vivo***
Fentanyl	n.d.	↓ (16–20)	↔↓(22, 36)	↓[POP](23–26) ↑[h.v.] (27)
Methadone	↓(33) ↔(30, 31)	↓ (34, 35) ↔ (32–34)	↓(36, 37) ↔ (36)	↔ [MMT] (39–44)
Oxycodone	↓ (50)	↔ (47–49)	n.d.	↓[POP] (53) ↔ [POP and cancer pain] (51–53)
Buprenorphine	n.d.	↔(16, 19, 56, 57)	↓↔ (59, 60)	↔ [BMT] (40, 41)
Remifentanil	n.d.	↓ (62, 63)	n.d.	↓ [POP](64, 65, 67) ↔ [POP](66)
Tramadol	n.d.	↑ (69–72)	↔(75)	↔↑[POP](73, 74)
Tapentadol	n.d.	↔(78)	n.d.	n.d.

*↓ significant decrease of at least one immune parameter*.

*↑significant increase of at least one immune parameter*.

*↔ no effect*.

*↓↔ different effect on different immune parameters*.

*n.d., not determined*.

*POP, peri–operative period*.

*h.v., healthy volunteers*.

*MMT, methadone maintenance treatment of opioid addicts*.

*BMT, buprenorphine maintenance treatment of opioid addicts*.

*() numbers in brackets are corresponding references*.

With some limitations and caution due to the different methods used to measure the responses of the immune system, the large range of the doses applied and the relative scarce number of participants in the available studies, we can conclude that it is not correct to generalize immunosuppression as a common side effect of all opioid molecules.

## Author Contributions

PS and SF undertook the literature search and contributed to study design, data collection, and data analysis. GM and GA contributed to the study design and critically read the manuscript.

### Conflict of Interest

The authors declare that the research was conducted in the absence of any commercial or financial relationships that could be construed as a potential conflict of interest.
